# Supplementary material for: Endocardial HDAC3 is required for myocardial trabeculation
Source: Nat Commun. 2024 May 16;15:4166. doi: 10.1038/s41467-024-48362-6 (PMC11099086; doi:10.1038/s41467-024-48362-6)
Supplement: Supplementary file 3 — Description of Additional Supplementary Files [file 41467_2024_48362_MOESM3_ESM.pdf]

Description of Additional Supplementary Files

Supplementary Data 1: Animal models, antibodies and qRT-PCR primers used in the study.
