# Supplementary material for: Endocardial HDAC3 is required for myocardial trabeculation
Source: Nat Commun. 2024 May 16;15:4166. doi: 10.1038/s41467-024-48362-6 (PMC11099086; doi:10.1038/s41467-024-48362-6)
Supplement: Supplementary file 5 — Reporting Summary [file 41467_2024_48362_MOESM5_ESM.pdf]

nature portfolio

Corresponding author(s): Deqiang Li

Last updated by author(s): Mar 24, 2024

Reporting Summary

Nature Portfolio wishes to improve the reproducibility of the work that we publish. This form provides structure for consistency and transparency in reporting. For further information on Nature Portfolio policies, see our Editorial Policies and the Editorial Policy Checklist.

Statistics

For all statistical analyses, confirm that the following items are present in the figure legend, table legend, main text, or Methods section.

n/a	Confirmed	
☐	☒	The exact sample size (*n*) for each experimental group/condition, given as a discrete number and unit of measurement
☐	☒	A statement on whether measurements were taken from distinct samples or whether the same sample was measured repeatedly
☐	☒	The statistical test(s) used AND whether they are one- or two-sided<br>*Only common tests should be described solely by name; describe more complex techniques in the Methods section.*
☒	☐	A description of all covariates tested
☐	☒	A description of any assumptions or corrections, such as tests of normality and adjustment for multiple comparisons
☐	☒	A full description of the statistical parameters including central tendency (e.g. means) or other basic estimates (e.g. regression coefficient) AND variation (e.g. standard deviation) or associated estimates of uncertainty (e.g. confidence intervals)
☐	☒	For null hypothesis testing, the test statistic (e.g. *F*, *t*, *r*) with confidence intervals, effect sizes, degrees of freedom and *P* value noted<br>*Give P values as exact values whenever suitable.*
☒	☐	For Bayesian analysis, information on the choice of priors and Markov chain Monte Carlo settings
☒	☐	For hierarchical and complex designs, identification of the appropriate level for tests and full reporting of outcomes
☒	☐	Estimates of effect sizes (e.g. Cohen's *d*, Pearson's *r*), indicating how they were calculated

Our web collection on statistics for biologists contains articles on many of the points above.

Software and code

Policy information about availability of computer code

Data collection	Imaging data were on a Zeiss LSM 710 confocal Microscope or a Leica DM6 fluorescence microscope. Epifluorescence and gross images of embryos were imaged on a Leica M205 FCA stereo fluorescence microscope. Western blots were developed using Series XXXV A Rapid Processor and scanned with EPSON PERFECTION 4870 PHOTO. Bioanalyzer (2100, Agilent) for analyzing the quality of the extracted RNA. qPCR data were collected using StepOne Plus Real-Time PCR system (Applied Biosystems). Bulk RNA-Seq and miRNA data were collected using Illumina NextSeq500 sequencer. scRNA-Seq data were collected using Illumina NextSeq S4.
Data analysis	Fiji software (National Institutes of Health [NIH]) was used to quantify all imaging data. Flowcytometry data analysis was performed using Flowjo software Ver 10.10. Graphpad Prism version 9.4.0 (Graph Pad Software) and Microsoft Excel version 16.72 (Microsoft) were used to analyze the data.<br>Bulk RNA-Seq and miRNA-Seq data: Data were analyzed using R package with "DESeq2" and "ClusterProfiler".<br>Single cell RNA sequencing (scRNA-Seq data): Reads were aligned to the Ensembl 104 mouse reference genome including the sequence for eYFP as a separate contig using STARsolo (version 2.7.10b). The resulting count tables (cells x genes) were converted into Annotated Data (anndata) format for analysis in Python. For quality control, all cells with less than 1500 or more than 9000 genes expressed were excluded. Additionally, cell doublets were filtered out using the Scrublet method. Gene counts were normalized per cell to a total target sum and transformed using log1p. Highly variable genes were identified using the Seurat method and these genes were utilized for dimension reduction with PCA. Finally, cell embedding was performed with the UMAP algorithm (umap-learn version 0.5.3) and cells were clustered using leiden (leidenalg version 0.9.1) with resolution 0.2. Scoring of trabecular and compact CM subtypes was performed using 'score_genes' and differentially expressed genes between groups were obtained using 'rank_genes_groups', both methods from the SCANPY package.

For manuscripts utilizing custom algorithms or software that are central to the research but not yet described in published literature, software must be made available to editors and reviewers. We strongly encourage code deposition in a community repository (e.g. GitHub). See the Nature Portfolio guidelines for submitting code & software for further information.

Data

Policy information about availability of data

All manuscripts must include a data availability statement. This statement should provide the following information, where applicable:

- Accession codes, unique identifiers, or web links for publicly available datasets
- A description of any restrictions on data availability
- For clinical datasets or third party data, please ensure that the statement adheres to our policy

All data associated with this study are present in the main text or the supplementary materials. Source data are provided with this paper. The scRNA-seq, RNA-seq and miRNA-seq data generated in this study have been deposited in the Gene Expression Omnibus under accession code GSE229661. All raw data are available from the authors upon reasonable request.

Research involving human participants, their data, or biological material

Policy information about studies with human participants or human data. See also policy information about sex, gender (identity/presentation), and sexual orientation and race, ethnicity and racism.

Reporting on sex and gender	N/A
Reporting on race, ethnicity, or other socially relevant groupings	N/A
Population characteristics	N/A
Recruitment	N/A
Ethics oversight	N/A

Note that full information on the approval of the study protocol must also be provided in the manuscript.

Field-specific reporting

Please select the one below that is the best fit for your research. If you are not sure, read the appropriate sections before making your selection.

☒ Life sciences ☐ Behavioural & social sciences ☐ Ecological, evolutionary & environmental sciences

For a reference copy of the document with all sections, see nature.com/documents/nr-reporting-summary-flat.pdf

Life sciences study design

All studies must disclose on these points even when the disclosure is negative.

Sample size	For animal studies, sample size is determined either by power calculation or with a minimum number of 6. For ex vivo quantifications, all tissues per biological replicate were included in the analysis. For in vitro studies, we typically conduct 4-8 technical replicates, depending on the accuracy of the assay, and according also to our previous experience (Jihyun Jang et al., 2022, Circulation Research, PMID: 35722872), in which significant changes can be consistently reproduced and are often biologically meaningful with such sample size.
Data exclusions	no data exclusions.
Replication	All experimental findings were replicated at least 3 times with enough reproducibility. All attempts at data replication were successful. Number of biological replicates in each panel is included in figure legends.
Randomization	All animals used in this manuscript were randomly assigned into each experimental group. For in vitro cell or cardiac explant culture experiments, cells and cardiac explants were allocated into each well and assigned with experimental conditions randomly.
Blinding	The researcher who performed histology and immunostaining was blinded to group allocation during the entire study. A researcher blinded to the group allocation was responsible for final data analysis.

Reporting for specific materials, systems and methods

We require information from authors about some types of materials, experimental systems and methods used in many studies. Here, indicate whether each material, system or method listed is relevant to your study. If you are not sure if a list item applies to your research, read the appropriate section before selecting a response.

Materials & experimental systems

n/a	Involved in the study
☐	☒ Antibodies
☐	☒ Eukaryotic cell lines
☒	☐ Palaeontology and archaeology
☐	☒ Animals and other organisms
☒	☐ Clinical data
☒	☐ Dual use research of concern
☒	☐ Plants

Methods

n/a	Involved in the study
☒	☐ ChIP-seq
☐	☒ Flow cytometry
☒	☐ MRI-based neuroimaging

Antibodies

Antibodies used

HDAC3 (Santa Cruz, sc-11417), IHC-1:50
CD31 (Dianova, Dia-310), IHC-1:200
BrdU (eBioscience, 14-5071-80) IHC-1:50
eNOS (BD Pharmingen, 610296) IHC-1:250
TNNT2 (Fisher Thermo Scientific, ms-295-P1) IHC-1:200
Ki67 (Invitrogen, MA5-14520) ICC-1:100
p-H3 (Cell Signaling, #9701S) IHC-1:20, ICC-1:100
GFP (Abcam, ab6673) IHC-1:200
VEGFb (GeneTex, GTX53099) WB-1:1,000
NRG1 (Proteintech, 10527-1-AP) WB-1:1,000
IGF1 (Origene, TA805792S) WB-1:1,000
CXCL12 (Abclonal, A18225) WB-1:1,000
TGFb1 (Proteintech, 21898-1-AP) WB-1:1,000
TGFb2 (Proteintech, 19999-1-AP) WB-1:1,000
TGFb3 (Invitrogen, PA5-78197) WB-1:1,000
GAPDH (Proteintech, HRP-60004) WB-1:10,000

Validation

All the antibodies used in this study were purchased from vendors and already validated by vendors.

HDAC3 (https://www.scbt.com/p/hdac3-antibody-h-99)
CD31 (IHC-1:200, https://www.arp1.com/anti-cd31-monoclonal-antibody-dia-310.html)
BrdU (IHC-1:50, https://www.thermofisher.com/antibody/product/BrdU-Antibody-clone-BU20A-Monoclonal/14-5071-37)
eNOS (https://www.bdbiosciences.com/en-us/products/reagents/microscopy-imaging-reagents/immunofluorescence-reagents/purified-mouse-anti-enos-nos-type-iii.610296)
TNNT2 (https://www.thermofisher.com/order/catalog/product/MS-295-P)
Ki67 (https://www.thermofisher.com/antibody/product/Ki-67-Antibody-clone-SP6-Monoclonal/MA5-14520)
p-H3 (https://www.cellsignal.com/products/primary-antibodies/phospho-histone-h3-ser10-antibody/9701?site-search-type=Products&N=4294956287&Ntt=9701s&fromPage=plp&_requestid=2130723)
GFP (https://www.abcam.com/gfp-antibody-ab6673.html)
VEGFb (https://www.genetex.com/Product/Detail/VEGFB-antibody-6E72/GTX53099?utm_source=Biocompare&utm_medium=referral&utm_campaign=Biocompare_GeneTex)
NRG1 (https://www.ptglab.com/products/NRG1-Antibody-10527-1-AP.htm)
IGF1 (https://www.origene.com/catalog/antibodies/primary-antibodies/ta805792s/igf1-mouse-monoclonal-antibody-clone-id-oti3a6)
CXCL12 (https://abclonal.com/catalog-antibodies/CXCL12RabbitpAb/A18225)
TGFb1 (https://www.ptglab.com/products/TGF-beta-1-Antibody-21898-1-AP.htm)
TGFb2 (https://www.ptglab.com/products/TGFB2-Specific-Antibody-19999-1-AP.htm)
TGFb3 (lhttps://www.bio-techne.com/p/antibodies/tgf-beta3-antibody-20724_mab243#technical-data-tab-tab)
GAPDH (https://www.ptglab.com/products/GAPDH-Antibody-HRP-60004.htm)

Eukaryotic cell lines

Policy information about cell lines and Sex and Gender in Research

Cell line source(s)	Mouse Cardiac Endotehlial Cells (MCECs) were purchased from Cedarlane labs (#CLU510).
Authentication	All cell lines were authenticated by STR profiling .
Mycoplasma contamination	All cell lines were routinely verified to be free of mycoplasma contamination.
Commonly misidentified lines (See ICLAC register)	No commonly misidentified cell lines were used in this study.

Animals and other research organisms

Policy information about studies involving animals; ARRIVE guidelines recommended for reporting animal research, and Sex and Gender in Research

Laboratory animals	Mice are all on C57BL/6J background. Mice of 8 weeks to 12 weeks old were used in this study. Tie2-Cre/+, Hdac3flox/+, and R26eYFP mice were purchased from the Jackson Laboratory (Stock numbers: #004128, #024119 and #006148, respectively). Nfatc1Cre/+ mice were provided by Bin Zhou lab at Albert Einstein College of Medicine. Tnnt2nGFP/+ mice were provided by Chen-Leng Cai lab at Indiana University School of Medicine.
Wild animals	No wild animals were used in this study
Reporting on sex	Both male and female mice are used in this study.
Field-collected samples	No field-collected samples were used in the study.
Ethics oversight	All animal protocols were approved by the Nationwide Children's Institutional Animal Care and Use Committee (IACUC #AR22-00194).

Note that full information on the approval of the study protocol must also be provided in the manuscript.

Plants

Seed stocks	N/A
Novel plant genotypes	N/A
Authentication	N/A

Flow Cytometry

Plots

Confirm that:

☒ The axis labels state the marker and fluorochrome used (e.g. CD4-FITC).

☒ The axis scales are clearly visible. Include numbers along axes only for bottom left plot of group (a 'group' is an analysis of identical markers).

☒ All plots are contour plots with outliers or pseudocolor plots.

☒ A numerical value for number of cells or percentage (with statistics) is provided.

Methodology

Sample preparation	E11.5 hearts were harvested in cold PBS, and then digested into single cells using 0.5% Trypsin-EDTA. the cells were washed with PBS, then resuspended in MACS buffer. Autofluorescence of YFP signal (using FITC channel) was used for detection and collection of cells.
Instrument	BD influx cell sorter
Software	Flowjo V10.
Cell population abundance	CECs are YFP positive cells.
Gating strategy	Live cells were select by FSC and SSC, and then analyzed by FITC.

☒ Tick this box to confirm that a figure exemplifying the gating strategy is provided in the Supplementary Information.
